# Supplementary material for: Site conditions for regeneration of climax species, the key for restoring moist deciduous tropical forest in Southern Vietnam
Source: PLoS One. 2020 May 29;15(5):e0233524. doi: 10.1371/journal.pone.0233524 (PMC7259571; doi:10.1371/journal.pone.0233524)
Supplement: S1 Data — (DOCX) [file pone.0233524.s001.docx]

**S1 Data collection methods**

**Leaf thickness, chlorophyll content and stomata density measure method**

The measurements of leaf size and chlorophyll content were conducted on 10-12 seedlings with a minimum total of 30 leaves for each treatment after ten months. The measured leaves were fully expanded and undamaged. For the low light level treatment, we measured all leaves of the surviving seedlings. Leaf length (L) was measured from the lamina tip to the point of petiole intersection along the midvein, while leaf width (W) was determined by the widest point across the leaf, perpendicular to the lamina midvein. Chlorophyll content was measured by using Opti‐Sciences, model CCM‐200 (Opti‐Sciences, Inc., Hudson, NH) at the centre (next to the midvein) of the leaf.

For each treatment we collected 180 leaf samples to measure leaf thickness (T) and stomata density (S). We measured leaf thickness (thickness) from leaf paradermal sections prepared as follows: was used. Leaf slides (25-35μm thick x 5 mm wide x 10-13 mm long) were taken from the middle portion of the lamina across the mid-rib at three portions of the leaf length by a sharp, double-sided razor. The leaf was then placed in commercial household bleach (Javel 10%) to remove the upper epidermis and palisade mesophyll (about 1’), rinsed in water, and replaced in water for c. 20 min to allow residual bleach to dissipate. The resulting section was stained with 1% toluidine blue for c. 2 min, rinsed gently but thoroughly in water, and mounted on microscope slides in phenol glycerine jelly. Leaf thickness were measured using Optica Vision Pro from digital photomicrographs of the paradermal sections at·x40 magnification.

Stomatal density (D.stomata) was measured on abaxial cuticles (stomata were absent from adaxial surfaces of leaves) prepared from the same leaves on which D.vein and leaf area were measured. The cuticles were prepared by dismounting the paradermal section by gently heating it on a hot plate, cutting it lengthways, rinsing one half in warm water to remove residual jelly, and then soaking in warm 10% aqueous Cr_2_O_3_ until clear, rinsing thoroughly, staining with dilute (< 0.1%) crystal violet, rinsing, if necessary cleaning with a single-hair paintbrush, and then mounting on microscope slides, in phenol glycerine jelly. The remaining half leaf was remounted for future reference. D.stomata was also measured on older cuticle preparations from the same populations. These preparations included three leaves from each tree from each population. The trees included all those from which measurements of D.vein were made. In all cases, stomatal densities were measured from digital photomicrographs of the cuticle preparation at ·50 magnification (giving 20–50 stomata per field of view) using the counting tool in IMAGEJ. At least three fields of view were measured from each section.

**References**

1. Frazer GW, Canham CD, Lertzman KP. Gap Light Analyzer (GLA): Imaging software to extract canopy structure and gap light transmission indices from true-colour fisheye photographs, users manual and program documentation. Simon Fraser University, Burnaby, British Columbia, and the Institute of Ecosystem Studies, Millbrook, New York.; 1999.

2. Nobis M. SideLook 1.1 - Imaging software for the analysis of vegetation structure with true-colour photographs. 2005.

3. Nobis M, Hunziker U. Automatic thresholding for hemispherical canopy-photographs based on edge detection. Agric For Meteorol. 2005;128: 243–250. doi:10.1016/j.agrformet.2004.10.002

4. Beven KJ, Kirkby MJ. A physically based, variable contributing area model of basin hydrology. Hydrol Sci Bull. 1979;24: 43–69. doi:10.1080/02626667909491834

5. Raduła MW, Szymura TH, Szymura M. Topographic wetness index explains soil moisture better than bioindication with Ellenberg’s indicator values. Ecol Indic. 2018;85: 172–179. doi:10.1016/j.ecolind.2017.10.011

6. Sørensen R, Zinko U, Seibert J. On the calculation of the topographic wetness index: Evaluation of different methods based on field observations. Hydrol Earth Syst Sci. 2006;10: 101–112. doi:10.5194/hess-10-101-2006
