# Supplementary material for: Site conditions for regeneration of climax species, the key for restoring moist deciduous tropical forest in Southern Vietnam
Source: PLoS One. 2020 May 29;15(5):e0233524. doi: 10.1371/journal.pone.0233524 (PMC7259571; doi:10.1371/journal.pone.0233524)
Supplement: S2 Data — (DOCX) [file pone.0233524.s002.docx]

**Data 1. Forest observation points data. All data at a observation points were follow the name of the image took at the point.**

| Image | openness | X | Y | Z | P.m | Soil | Plot | BA | PD.A | Stand.S.R | Dyeri.P.A | dyeri.S | TWI | slope |
| --- | --- | --- | --- | --- | --- | --- | --- | --- | --- | --- | --- | --- | --- | --- |
| 2208 | 14.75 | 1158 | 6514 | 68 | O.Al | Fp | SS3 | 35.7 | 0.4 | 31 | 1 | 1 | 12.2 | 2 |
| 2209 | 10.06 | 1168 | 6521 | 68 | O.Al | Fp | SS3 | 35.7 | 0.4 | 31 | 1 | 1 | 10.8 | 2 |
| 2217 | 11.88 | 1141 | 6616 | 68 | O.Al | Fp | SS3 | 35.7 | 0.4 | 31 | 1 | 1 | 7.4 | 2 |
| 2219 | 12.1 | 1132 | 6605 | 68 | O.Al | Fp | SS3 | 35.7 | 0.4 | 31 | 1 | 1 | 7.4 | 2 |
| 2221 | 14.04 | 1133 | 6610 | 68 | O.Al | Fp | SS3 | 35.7 | 0.4 | 31 | 1 | 1 | 7.4 | 2 |
| 2225 | 7.05 | 1136 | 6595 | 66 | O.Al | Fp | SS3 | 35.7 | 0.4 | 31 | 1 | 2 | 7.4 | 2 |
| 2258 | 7.53 | 1119 | 6611 | 68 | O.Al | Fp | SS3 | 35.7 | 0.4 | 31 | 1 | 1 | 8.5 | 2 |
| 2259 | 7.87 | 1115 | 6613 | 68 | O.Al | Fp | SS3 | 35.7 | 0.4 | 31 | 1 | 1 | 8.5 | 2 |
| 2261 | 7.69 | 1117 | 6623 | 68 | O.Al | Fp | SS3 | 35.7 | 0.4 | 31 | 1 | 2 | 7.4 | 2 |
| 2263 | 8.7 | 1117 | 6634 | 68 | O.Al | Fp | SS3 | 35.7 | 0.4 | 31 | 1 | 1 | 7.4 | 2 |
| 2264 | 8.19 | 1114 | 6648 | 69 | O.Al | Fp | SS3 | 35.7 | 0.4 | 31 | 1 | 1 | 7.4 | 2 |
| 2267 | 9.19 | 1120 | 6652 | 69 | O.Al | Fp | SS3 | 35.7 | 0.4 | 31 | 1 | 1 | 7.0 | 2 |
| 2303 | 15.06 | 2505 | 652 | 73 | O.Al | Fp | RR1 | 42.2 | 0.8 | 5 | 1 | 1 | 10.3 | 2 |
| 2314 | 13.81 | 2566 | 773 | 72 | O.Al | Fp | RR1 | 42.2 | 0.8 | 5 | 1 | 4 | 6.7 | 2 |
| 2315 | 15.56 | 2581 | 779 | 72 | O.Al | Fp | RR1 | 42.2 | 0.8 | 5 | 1 | 3 | 6.7 | 2 |
| 2316 | 15.1 | 2595 | 775 | 73 | O.Al | Fp | RR1 | 42.2 | 0.8 | 5 | 1 | 3 | 6.7 | 2 |
| 2319 | 7.49 | 2609 | 779 | 73 | O.Al | Fp | RR3 | 26.9 | 0.2 | 34 | 1 | 2 | 6.6 | 2 |
| 2321 | 7.29 | 2625 | 783 | 73 | O.Al | Fp | RR3 | 26.9 | 0.2 | 34 | 1 | 2 | 6.6 | 2 |
| 2323 | 8.94 | 2477 | 679 | 73 | O.Al | Fp | RR3 | 26.9 | 0.2 | 34 | 1 | 1 | 8.7 | 2 |
| 2325 | 6.91 | 2475 | 697 | 73 | O.Al | Fp | RR3 | 26.9 | 0.2 | 34 | 1 | 1 | 7.7 | 2 |
| 2369 | 7.89 | 2563 | 777 | 72 | O.Al | Fp | RR3 | 26.9 | 0.2 | 34 | 1 | 2 | 6.8 | 2 |
| 2374 | 15.29 | 2647 | 770 | 73 | O.Al | Fp | RR2 | 35.7 | 0.4 | 31 | 1 | 1 | 6.9 | 2 |
| 2376 | 17.81 | 2667 | 768 | 73 | O.Al | Fp | RR2 | 35.7 | 0.4 | 31 | 1 | 1 | 6.9 | 2 |
| 2378 | 18.28 | 2659 | 775 | 73 | O.Al | Fp | RR2 | 35.7 | 0.4 | 31 | 1 | 1 | 6.6 | 2 |
| 2397 | 18.91 | 2666 | 1064 | 73 | O.Al | Fp | RR2 | 35.7 | 0.4 | 31 | 1 | 1 | 9.9 | 2 |
| 2399 | 14.12 | 2663 | 1051 | 73 | O.Al | Fp | RR2 | 35.7 | 0.4 | 31 | 1 | 1 | 9.9 | 2 |
| 2401 | 8.58 | 2669 | 1053 | 73 | O.Al | Fp | RR2 | 35.7 | 0.4 | 31 | 1 | 1 | 9.9 | 2 |
| 2403 | 10.48 | 2668 | 1055 | 73 | O.Al | Fp | RR2 | 35.7 | 0.4 | 31 | 1 | 1 | 9.9 | 2 |
| 2405 | 19.09 | 6166 | 6066 | 52 | O.Al | Fp | DD1 | 19.6 | 0.2 | 29 | 1 | 1 | 7.2 | 11 |
| 2407 | 17.14 | 6230 | 6083 | 53 | O.Al | Fp | DD1 | 19.6 | 0.2 | 29 | 1 | 1 | 7.0 | 11 |
| 2420 | 17.64 | 6233 | 6091 | 54 | O.Al | Fp | DD1 | 19.6 | 0.2 | 29 | 1 | 1 | 7.0 | 11 |
| 2421 | 12.76 | 6236 | 6091 | 54 | O.Al | Fp | DD1 | 19.6 | 0.2 | 29 | 1 | 1 | 7.0 | 11 |
| 2423 | 12.61 | 6232 | 6097 | 54 | O.Al | Fp | DD1 | 19.6 | 0.2 | 29 | 1 | 1 | 7.0 | 11 |
| 2424 | 9.81 | 6234 | 6101 | 54 | O.Al | Fp | DD1 | 19.6 | 0.2 | 29 | 1 | 1 | 7.0 | 11 |
| 2426 | 8.4 | 6239 | 6101 | 54 | O.Al | Fp | DD2 | 13.6 | 0.0 | 30 | 1 | 1 | 7.1 | 11 |
| 2428 | 11.44 | 6245 | 6115 | 54 | O.Al | Fp | DD2 | 13.6 | 0.0 | 30 | 1 | 1 | 7.1 | 11 |
| 2429 | 11.63 | 6242 | 6112 | 54 | O.Al | Fp | DD2 | 13.6 | 0.0 | 30 | 1 | 1 | 7.1 | 11 |
| 2431 | 15.42 | 6233 | 6141 | 58 | O.Al | Fp | DD2 | 13.6 | 0.0 | 30 | 1 | 1 | 7.1 | 11 |
| 2433 | 15.44 | 6238 | 6121 | 54 | O.Al | Fp | DD2 | 13.6 | 0.0 | 30 | 1 | 1 | 7.0 | 11 |
| 2435 | 9.3 | 6262 | 6131 | 59 | O.Al | Fp | DD2 | 13.6 | 0.0 | 30 | 0 | 0 | 7.6 | 11 |
| 2438 | 8.13 | 6240 | 6142 | 58 | O.Al | Fp | DD2 | 13.6 | 0.0 | 30 | 1 | 1 | 7.6 | 11 |
| 2440 | 8.54 | 6233 | 6142 | 58 | O.Al | Fp | DD2 | 13.6 | 0.0 | 30 | 1 | 1 | 7.1 | 11 |
| 2442 | 9.63 | 6241 | 6137 | 58 | O.Al | Fp | DD2 | 13.6 | 0.0 | 30 | 1 | 1 | 7.6 | 11 |
| 2444 | 8.34 | 6236 | 6142 | 58 | O.Al | Fp | DD2 | 13.6 | 0.0 | 30 | 1 | 2 | 7.1 | 11 |
| 2450 | 9.76 | 6265 | 6132 | 59 | O.Al | Fp | DD2 | 13.6 | 0.0 | 30 | 1 | 1 | 7.6 | 11 |
| 2452 | 10.81 | 6268 | 6126 | 59 | O.Al | Fp | DD2 | 13.6 | 0.0 | 30 | 1 | 1 | 7.1 | 11 |
| 2454 | 9.23 | 6268 | 6122 | 59 | O.Al | Fp | DD2 | 13.6 | 0.0 | 30 | 1 | 1 | 7.1 | 11 |
| 2484 | 8.79 | 6343 | 6140 | 56 | O.Al | Fp | DD2 | 13.6 | 0.0 | 30 | 1 | 1 | 8.5 | 11 |
| 2486 | 8.55 | 5692 | 6198 | 50 | O.Al | Fp | DDHR1 | 50.2 | 0.2 | 24 | 1 | 1 | 6.2 | 14 |
| 2519 | 10.39 | 5691 | 6218 | 51 | O.Al | Fp | DDHR1 | 50.2 | 0.2 | 24 | 1 | 1 | 5.8 | 14 |
| 2521 | 14.05 | 5694 | 6223 | 51 | O.Al | Fp | DDHR1 | 50.2 | 0.2 | 24 | 1 | 1 | 5.8 | 13 |
| 2523 | 12.72 | 5698 | 6241 | 51 | O.Al | Fp | DDHR1 | 50.2 | 0.2 | 24 | 1 | 1 | 5.8 | 10 |
| 2525 | 9.56 | 5691 | 6236 | 51 | O.Al | Fp | DDHR1 | 50.2 | 0.2 | 24 | 0 | 0 | 5.8 | 13 |
| 2526 | 11.6 | 5690 | 6251 | 53 | O.Al | Fp | DDHR1 | 50.2 | 0.2 | 24 | 0 | 0 | 5.7 | 12 |
| 2527 | 20.78 | 5679 | 6242 | 51 | O.Al | Fp | DDHR1 | 50.2 | 0.2 | 24 | 0 | 0 | 6.4 | 13 |
| 2599 | 19.21 | 4881 | 5936 | 50 | O.Al | Fp | DDDY4 | 23.4 | 0.3 | 26 | 0 | 0 | 7.1 | 20 |
| 2601 | 15.14 | 4874 | 5959 | 50 | O.Al | Fp | DDDY4 | 23.4 | 0.3 | 26 | 0 | 0 | 7.3 | 18 |
| 2603 | 18.01 | 4866 | 5952 | 50 | O.Al | Fp | DDDY4 | 23.4 | 0.3 | 26 | 0 | 0 | 7.3 | 26 |
| 2606 | 13.7 | 4877 | 5942 | 50 | O.Al | Fp | DDDY4 | 23.4 | 0.3 | 26 | 0 | 0 | 7.3 | 24 |
| 2608 | 11.69 | 4872 | 5930 | 50 | O.Al | Fp | DDDY4 | 23.4 | 0.3 | 26 | 0 | 0 | 7.1 | 24 |
| 2610 | 11.83 | 4930 | 5989 | 59 | O.Al | Fp | DDDY4 | 23.4 | 0.3 | 26 | 1 | 1 | 6.9 | 18 |
| 2612 | 14.56 | 4883 | 5949 | 50 | O.Al | Fp | DDDY4 | 23.4 | 0.3 | 26 | 1 | 2 | 7.3 | 18 |
| 2637 | 14.99 | 5254 | 6174 | 44 | O.Al | Fp | DDHR1 | 50.2 | 0.2 | 24 | 0 | 0 | 5.1 | 8 |
| 2639 | 14.63 | 5266 | 6180 | 44 | O.Al | Fp | DDHR1 | 50.2 | 0.2 | 24 | 0 | 0 | 5.8 | 8 |
| 2641 | 14.65 | 5250 | 6168 | 44 | O.Al | Fp | DDHR1 | 50.2 | 0.2 | 24 | 0 | 0 | 5.1 | 8 |
| 2643 | 14.96 | 5247 | 6192 | 44 | O.Al | Fp | DDHR1 | 50.2 | 0.2 | 24 | 0 | 0 | 5.2 | 26 |
| 2645 | 19.8 | 5264 | 6190 | 44 | O.Al | Fp | DDHR1 | 50.2 | 0.2 | 24 | 1 | 1 | 5.9 | 31 |
| 2647 | 17.6 | 5242 | 6201 | 44 | O.Al | Fp | DDHR1 | 50.2 | 0.2 | 24 | 0 | 0 | 5.2 | 29 |
| 2689 | 15.13 | 5240 | 6220 | 44 | O.Al | Fp | DDHR1 | 50.2 | 0.2 | 24 | 1 | 5 | 5.8 | 13 |
| 2691 | 16.17 | 5240 | 6243 | 44 | O.Al | Fp | DDHR1 | 50.2 | 0.2 | 24 | 1 | 8 | 5.8 | 10 |
| 2692 | 11.17 | 6463 | 840 | 76 | O.Al | Fp | CG1 | 34.1 | 0.3 | 25 | 0 | 0 | 9.6 | 5 |
| 2695 | 18.59 | 6471 | 837 | 76 | O.Al | Fp | CG1 | 34.1 | 0.3 | 25 | 0 | 0 | 9.6 | 5 |
| 2696 | 18.81 | 6446 | 846 | 75 | O.Al | Fp | CG1 | 34.1 | 0.3 | 25 | 0 | 0 | 7.8 | 5 |
| 2697 | 18.28 | 6449 | 856 | 77 | O.Al | Fp | CG1 | 34.1 | 0.3 | 25 | 1 | 2 | 7.8 | 5 |
| 2698 | 15.52 | 6461 | 850 | 77 | O.Al | Fp | CG1 | 34.1 | 0.3 | 25 | 1 | 3 | 9.6 | 5 |
| 2743 | 14.49 | 6516 | 773 | 77 | O.Al | Fp | CG1 | 34.1 | 0.3 | 25 | 1 | 11 | 8.2 | 5 |
| 2775 | 10.95 | 6759 | 183 | 75 | O.Al | Fp | CG2 | 32.7 | 0.0 | 25 | 0 | 0 | 8.3 | 5 |
| 2778 | 10.44 | 6747 | 192 | 76 | O.Al | Fp | CG2 | 32.7 | 0.0 | 25 | 0 | 0 | 8.2 | 5 |
| 2780 | 14.55 | 6716 | 168 | 80 | O.Al | Fp | CG2 | 32.7 | 0.0 | 25 | 0 | 0 | 8.5 | 5 |
| 2782 | 12.98 | 6714 | 151 | 80 | O.Al | Fp | CG2 | 32.7 | 0.0 | 25 | 0 | 0 | 7.6 | 5 |
| 2784 | 16.11 | 4282 | 4461 | 85 | O.Al | Fp | CG2 | 32.7 | 0.0 | 25 | 0 | 0 | 7.3 | 5 |
| 2829 | 14.2 | 5676 | 9781 | 80 | O.Al | Fp | RGHR | 44.0 | 0.5 | 30 | 0 | 0 | 6.2 | 5 |
| 2831 | 14.44 | 5656 | 9769 | 80 | O.Al | Fp | RGHR | 44.0 | 0.5 | 30 | 0 | 0 | 6.2 | 5 |
| 2833 | 12.44 | 5681 | 9768 | 80 | O.Al | Fp | RGHR | 44.0 | 0.5 | 30 | 1 | 1 | 6.2 | 5 |
| 2835 | 14.6 | 5706 | 9766 | 80 | O.Al | Fp | RGHR | 44.0 | 0.5 | 30 | 0 | 0 | 6.4 | 5 |
| 2838 | 13.55 | 5713 | 9770 | 80 | O.Al | Fp | RGHR | 44.0 | 0.5 | 30 | 1 | 1 | 6.4 | 5 |
| 2875 | 8.28 | 2617 | 7243 | 72 | O.Al | Fs | Km9.1 | 35.6 | 0.1 | 26 | 1 | 1 | 8.2 | 3 |
| 2895 | 11.87 | 2619 | 7242 | 72 | O.Al | Fs | Km9.1 | 35.6 | 0.1 | 26 | 0 | 0 | 8.2 | 3 |
| 2897 | 11.23 | 2624 | 7236 | 74 | O.Al | Fs | Km9.1 | 35.6 | 0.1 | 26 | 0 | 0 | 8.2 | 3 |
| 2899 | 13.86 | 2632 | 7236 | 74 | O.Al | Fs | Km9.1 | 35.6 | 0.1 | 26 | 0 | 0 | 8.2 | 3 |
| 2970 | 11.53 | 7103 | 7905 | 123 | Schist | Fk | SK1 | 15.4 | 0.1 | 26 | 0 | 0 | 7.3 | 7 |
| 2972 | 24.92 | 7097 | 7904 | 123 | Schist | Fk | SK1 | 15.4 | 0.1 | 26 | 0 | 0 | 7.2 | 7 |
| 2974 | 19.02 | 7081 | 7899 | 123 | Schist | Fk | SK1 | 15.4 | 0.1 | 26 | 0 | 0 | 7.2 | 7 |
| 2976 | 20.9 | 7073 | 7895 | 123 | Schist | Fk | SK1 | 15.4 | 0.1 | 26 | 0 | 0 | 7.2 | 7 |
| 2978 | 24.55 | 7071 | 7895 | 123 | Schist | Fk | SK1 | 15.4 | 0.1 | 26 | 0 | 0 | 6.8 | 8 |
| 2996 | 19.52 | 7057 | 7654 | 121 | Schist | Fk | SK1 | 15.4 | 0.1 | 26 | 1 | 1 | 7.8 | 25 |
| 2998 | 16.77 | 6469 | 7833 | 120 | Schist | Fk | SK1 | 15.4 | 0.1 | 26 | 1 | 1 | 6.9 | 32 |
| 3010 | 11.08 | 6613 | 6820 | 57 | O.Al | Fp | DD3 | 49.6 | 0.4 | 20 | 0 | 0 | 6.8 | 21 |
| 3012 | 15.31 | 6608 | 6850 | 57 | O.Al | Fp | DD3 | 49.6 | 0.4 | 20 | 1 | 2 | 7.0 | 22 |
| 3013 | 12.01 | 6605 | 6850 | 57 | O.Al | Fp | DD3 | 49.6 | 0.4 | 20 | 1 | 1 | 7.0 | 31 |
| 3014 | 16.28 | 6602 | 6860 | 57 | O.Al | Fp | DD3 | 49.6 | 0.4 | 20 | 0 | 0 | 7.0 | 18 |
| 3015 | 14.92 | 6593 | 6840 | 57 | O.Al | Fp | DD3 | 49.6 | 0.4 | 20 | 1 | 5 | 7.0 | 18 |
| 3060 | 15.32 | 7482 | 8290 | 130 | Schist | Fk | SK2 | 9.7 | 0.3 | 5 | 1 | 1 | 6.1 | 9 |
| 3062 | 10.9 | 7485 | 8300 | 129 | Schist | Fk | SK2 | 9.7 | 0.3 | 5 | 0 | 0 | 6.1 | 11 |
| 3063 | 23.96 | 7496 | 8290 | 130 | Schist | Fk | SK2 | 9.7 | 0.3 | 5 | 0 | 0 | 6.1 | 14 |
| 3064 | 10 | 7500 | 8300 | 129 | Schist | Fk | SK2 | 9.7 | 0.3 | 5 | 0 | 0 | 6.1 | 17 |
| 3066 | 11.91 | 7509 | 8290 | 130 | Schist | Fk | SK2 | 9.7 | 0.3 | 5 | 0 | 0 | 6.9 | 17 |
| 3067 | 7.74 | 7510 | 8290 | 130 | Schist | Fk | SK2 | 9.7 | 0.3 | 5 | 1 | 1 | 6.9 | 16 |
| 3068 | 11.9 | 7492 | 8300 | 129 | Schist | Fk | SK2 | 9.7 | 0.3 | 5 | 1 | 1 | 6.1 | 18 |
| 3074 | 23.43 | 6570 | 7930 | 123 | Schist | Fk | SK3 | 3.0 | 0.3 | 7 | 1 | 1 | 7.5 | 15 |
| 3076 | 16.58 | 6577 | 7950 | 123 | Schist | Fk | SK3 | 3.0 | 0.3 | 7 | 1 | 1 | 8.6 | 14 |
| 3078 | 15.14 | 6575 | 7950 | 123 | Schist | Fk | SK3 | 3.0 | 0.3 | 7 | 0 | 0 | 8.6 | 3 |
| 3079 | 13.69 | 6597 | 7940 | 123 | Schist | Fk | SK3 | 3.0 | 0.3 | 7 | 1 | 1 | 7.1 | 4 |
| 3080 | 14.04 | 6581 | 7920 | 123 | Schist | Fk | SK3 | 3.0 | 0.3 | 7 | 0 | 0 | 7.1 | 7 |
| 3081 | 14.92 | 6577 | 7930 | 123 | Schist | Fk | SK3 | 3.0 | 0.3 | 7 | 0 | 0 | 7.5 | 4 |
| 3082 | 16.02 | 6581 | 7940 | 123 | Schist | Fk | SK3 | 3.0 | 0.3 | 7 | 1 | 1 | 7.1 | 4 |
| 3083 | 17.14 | 1646 | 7510 | 83 | O.Al | Fp | Km9.2 | 35.4 | 0.3 | 17 | 0 | 0 | 7.1 | 4 |
| 3084 | 15.56 | 1673 | 7500 | 81 | O.Al | Fp | Km9.2 | 35.4 | 0.3 | 17 | 0 | 0 | 6.8 | 3 |
| 3085 | 13 | 1661 | 7500 | 83 | O.Al | Fp | Km9.2 | 35.4 | 0.3 | 17 | 0 | 0 | 6.8 | 3 |
| 3086 | 7.5 | 1653 | 7490 | 83 | O.Al | Fp | Km9.2 | 35.4 | 0.3 | 17 | 0 | 0 | 7.1 | 3 |
| 3087 | 13.76 | 1641 | 7500 | 83 | O.Al | Fp | Km9.2 | 35.4 | 0.3 | 17 | 1 | 1 | 7.1 | 3 |
| 3088 | 12.58 | 1643 | 7490 | 83 | O.Al | Fp | Km9.2 | 35.4 | 0.3 | 17 | 1 | 4 | 7.1 | 3 |

**Data 2. Observation point soil characteristics**

| Image | texture | color.m | colour | nodule | litter | litter.p | Topsoil.BD | pH | OC | N | CN | P2O5 | CEC | Sand | Fine.sand | Silt |
| --- | --- | --- | --- | --- | --- | --- | --- | --- | --- | --- | --- | --- | --- | --- | --- | --- |
| 2208 | LMC | 10YR6.6 | Brownish yellow | 50 | 4 | 50 | 1.2 | 4.0 | 2.0 | 0.2 | 9.6 | 7.5 | 7.8 | 23.6 | 14.8 | 48.0 |
| 2209 | MC | 10YR7.6 | Yellow | 30 | 2 | 30 | 1.1 | 4.0 | 1.7 | 0.2 | 9.3 | 5.7 | 7.1 | 26.1 | 18.1 | 44.2 |
| 2217 | HC | 10YR6.6 | Brownish yellow | 70 | 4 | 70 | 1.4 | 3.9 | 1.7 | 0.2 | 9.0 | 6.6 | 6.8 | 32.9 | 21.1 | 34.0 |
| 2219 |  |  |  |  | 2 | 80 | 1.2 | 4.0 | 1.8 | 0.2 | 9.3 | 6.6 | 7.2 | 27.6 | 18.0 | 42.1 |
| 2221 |  |  |  |  | 5 | 90 | 1.2 | 4.0 | 1.8 | 0.2 | 9.3 | 6.6 | 7.2 | 27.6 | 18.0 | 42.1 |
| 2225 |  |  |  |  | 4 | 50 | 1.2 | 4.0 | 1.8 | 0.2 | 9.3 | 6.6 | 7.2 | 27.6 | 18.0 | 42.1 |
| 2258 |  |  |  |  |  |  | 1.2 | 4.0 | 1.8 | 0.2 | 9.3 | 6.6 | 7.2 | 27.6 | 18.0 | 42.1 |
| 2259 |  |  |  |  |  |  | 1.2 | 4.0 | 1.8 | 0.2 | 9.3 | 6.6 | 7.2 | 27.6 | 18.0 | 42.1 |
| 2261 |  |  |  |  |  |  | 1.2 | 4.0 | 1.8 | 0.2 | 9.3 | 6.6 | 7.2 | 27.6 | 18.0 | 42.1 |
| 2263 |  |  |  |  |  |  | 1.2 | 4.0 | 1.8 | 0.2 | 9.3 | 6.6 | 7.2 | 27.6 | 18.0 | 42.1 |
| 2264 |  |  |  |  |  |  | 1.2 | 4.0 | 1.8 | 0.2 | 9.3 | 6.6 | 7.2 | 27.6 | 18.0 | 42.1 |
| 2267 |  |  |  |  |  |  | 1.2 | 4.0 | 1.8 | 0.2 | 9.3 | 6.6 | 7.2 | 27.6 | 18.0 | 42.1 |
| 2303 | LMC | 10YR6.8 | Brownish yellow | 7 | 2 | 50 | 1.2 | 3.5 | 2.1 | 0.2 | 9.6 | 6.4 | 8.1 | 9.3 | 19.1 | 51.2 |
| 2314 |  |  |  |  |  |  | 1.2 | 3.5 | 2.1 | 0.2 | 9.6 | 6.4 | 8.1 | 9.3 | 19.1 | 51.2 |
| 2315 |  |  |  |  |  |  | 1.2 | 3.5 | 2.1 | 0.2 | 9.6 | 6.4 | 8.1 | 9.3 | 19.1 | 51.2 |
| 2316 |  |  |  |  | 2 | 15 | 1.2 | 3.5 | 2.1 | 0.2 | 9.6 | 6.4 | 8.1 | 9.3 | 19.1 | 51.2 |
| 2319 | LMC | 10YR6.6 | Brownish yellow | 2 | 1 | 15 |  | 3.5 | 2.8 | 0.2 | 11.2 | 7.1 | 10.1 | 12.0 | 19.6 | 43.4 |
| 2321 |  |  |  |  | 3 | 30 |  | 3.5 | 2.8 | 0.2 | 11.2 | 7.1 | 10.1 | 12.0 | 19.6 | 43.4 |
| 2323 |  |  |  |  | 4 | 10 |  | 3.5 | 2.8 | 0.2 | 11.2 | 7.1 | 10.1 | 12.0 | 19.6 | 43.4 |
| 2325 |  |  |  |  | 2 | 5 |  | 3.5 | 2.8 | 0.2 | 11.2 | 7.1 | 10.1 | 12.0 | 19.6 | 43.4 |
| 2369 |  |  |  |  |  |  |  | 3.5 | 2.8 | 0.2 | 11.2 | 7.1 | 10.1 | 12.0 | 19.6 | 43.4 |
| 2374 |  |  |  |  |  |  | 0.6 | 3.8 | 0.9 | 0.1 | 9.3 | 3.2 | 3.4 | 10.8 | 9.0 | 18.2 |
| 2376 | MC | 10YR5.6 | Yellowish brown | 30 | 3 | 15 | 1.2 | 3.8 | 1.8 | 0.2 | 9.3 | 6.4 | 6.8 | 21.6 | 18.0 | 36.4 |
| 2378 |  |  |  |  |  |  | 0.6 | 3.8 | 0.9 | 0.1 | 9.3 | 3.2 | 3.4 | 10.8 | 9.0 | 18.2 |
| 2397 |  |  |  |  |  |  | 0.6 | 3.8 | 0.9 | 0.1 | 9.3 | 3.2 | 3.4 | 10.8 | 9.0 | 18.2 |
| 2399 | MC | 2.5YR7.4 | Light reddish brown | 30 | 2 | 25 | 0.9 | 3.9 | 2.3 | 0.3 | 9.2 | 6.4 | 6.1 | 21.1 | 22.9 | 46.0 |
| 2401 |  |  |  |  | 5 | 50 | 0.6 | 3.8 | 0.9 | 0.1 | 9.3 | 3.2 | 3.4 | 10.8 | 9.0 | 18.2 |
| 2403 |  |  |  |  | 2 | 15 | 0.6 | 3.8 | 0.9 | 0.1 | 9.3 | 3.2 | 3.4 | 10.8 | 9.0 | 18.2 |
| 2405 | MC | 10YR4.6 | Dark yellowes brown | 20 | 2 | 25 | 1.0 | 3.6 | 4.1 | 0.3 | 13.5 | 8.5 | 11.1 | 24.6 | 17.6 | 1.8 |
| 2407 |  |  |  |  | 1 | 5 | 1.0 | 3.7 | 4.1 | 0.3 | 13.7 | 9.9 | 10.2 | 23.4 | 14.9 | 11.6 |
| 2420 |  |  |  |  | 2 | 10 | 1.0 | 3.7 | 4.1 | 0.3 | 13.7 | 9.9 | 10.2 | 23.4 | 14.9 | 11.6 |
| 2421 | MC | 5YR5.6 | Yellowish red | 10 | 1 | 5 | 1.1 | 3.7 | 4.1 | 0.3 | 13.9 | 11.2 | 9.3 | 22.1 | 12.3 | 21.4 |
| 2423 |  |  |  |  | 1 | 5 | 1.0 | 3.7 | 4.1 | 0.3 | 13.7 | 9.9 | 10.2 | 23.4 | 14.9 | 11.6 |
| 2424 |  |  |  |  | 1 | 10 | 1.0 | 3.7 | 4.1 | 0.3 | 13.7 | 9.9 | 10.2 | 23.4 | 14.9 | 11.6 |
| 2426 |  |  |  |  | 1 | 10 | 1.1 | 3.7 | 4.1 | 0.3 | 13.9 | 11.2 | 9.3 | 22.1 | 12.3 | 21.4 |
| 2428 |  |  |  |  | 1 | 5 | 1.1 | 3.7 | 4.1 | 0.3 | 13.9 | 11.2 | 9.3 | 22.1 | 12.3 | 21.4 |
| 2429 |  |  |  |  | 1 | 5 | 1.1 | 3.7 | 4.1 | 0.3 | 13.9 | 11.2 | 9.3 | 22.1 | 12.3 | 21.4 |
| 2431 |  |  |  |  | 1 | 10 | 1.1 | 3.7 | 4.1 | 0.3 | 13.9 | 11.2 | 9.3 | 22.1 | 12.3 | 21.4 |
| 2433 |  |  |  |  | 1 | 5 | 1.1 | 3.7 | 4.1 | 0.3 | 13.9 | 11.2 | 9.3 | 22.1 | 12.3 | 21.4 |
| 2435 |  |  |  |  | 5 | 15 | 1.1 | 3.7 | 4.1 | 0.3 | 13.9 | 11.2 | 9.3 | 22.1 | 12.3 | 21.4 |
| 2438 |  |  |  |  | 1 | 7 | 1.1 | 3.7 | 4.1 | 0.3 | 13.9 | 11.2 | 9.3 | 22.1 | 12.3 | 21.4 |
| 2440 |  |  |  |  |  |  | 1.1 | 3.7 | 4.1 | 0.3 | 13.9 | 11.2 | 9.3 | 22.1 | 12.3 | 21.4 |
| 2442 |  |  |  |  |  |  | 1.1 | 3.7 | 4.1 | 0.3 | 13.9 | 11.2 | 9.3 | 22.1 | 12.3 | 21.4 |
| 2444 |  |  |  |  | 1 | 5 | 1.1 | 3.7 | 4.1 | 0.3 | 13.9 | 11.2 | 9.3 | 22.1 | 12.3 | 21.4 |
| 2450 |  |  |  |  | 1 | 5 | 1.1 | 3.7 | 4.1 | 0.3 | 13.9 | 11.2 | 9.3 | 22.1 | 12.3 | 21.4 |
| 2452 |  |  |  |  | 1 | 5 | 1.1 | 3.7 | 4.1 | 0.3 | 13.9 | 11.2 | 9.3 | 22.1 | 12.3 | 21.4 |
| 2454 |  |  |  |  | 1 | 5 | 1.1 | 3.7 | 4.1 | 0.3 | 13.9 | 11.2 | 9.3 | 22.1 | 12.3 | 21.4 |
| 2484 |  |  |  |  |  |  | 1.1 | 3.7 | 4.1 | 0.3 | 13.9 | 11.2 | 9.3 | 22.1 | 12.3 | 21.4 |
| 2486 |  |  |  |  |  | 60 | 1.4 | 4.0 | 1.9 | 0.2 | 9.2 | 7.1 | 6.5 | 58.4 | 20.0 | 12.4 |
| 2519 |  |  |  |  |  | 15 | 1.4 | 4.0 | 1.9 | 0.2 | 9.2 | 7.1 | 6.5 | 58.4 | 20.0 | 12.4 |
| 2521 | SCL | 10RY 5.6 | Yellowish brown | 20 | 2 | 7 | 1.4 | 4.0 | 1.9 | 0.2 | 9.2 | 7.1 | 6.5 | 58.4 | 20.0 | 12.4 |
| 2523 |  |  |  |  | 4 | 50 | 1.4 | 4.0 | 1.9 | 0.2 | 9.2 | 7.1 | 6.5 | 58.4 | 20.0 | 12.4 |
| 2525 |  |  |  |  | 5 | 50 | 1.4 | 4.0 | 1.9 | 0.2 | 9.2 | 7.1 | 6.5 | 58.4 | 20.0 | 12.4 |
| 2526 |  |  |  |  | 3 | 25 | 1.4 | 4.0 | 1.9 | 0.2 | 9.2 | 7.1 | 6.5 | 58.4 | 20.0 | 12.4 |
| 2527 |  |  |  |  | 6 | 90 | 1.4 | 4.0 | 1.9 | 0.2 | 9.2 | 7.1 | 6.5 | 58.4 | 20.0 | 12.4 |
| 2599 | LMC | 10YR5.6 | Yellowish brown | 25 |  |  | 0.9 | 4.2 | 3.9 | 0.3 | 11.6 | 10.0 | 14.7 | 16.3 | 36.9 | 30.6 |
| 2601 |  |  |  |  | 5 | 45 | 0.9 | 4.2 | 3.9 | 0.3 | 11.6 | 10.0 | 14.7 | 16.3 | 36.9 | 30.6 |
| 2603 |  |  |  |  | 8 | 70 | 0.9 | 4.2 | 3.9 | 0.3 | 11.6 | 10.0 | 14.7 | 16.3 | 36.9 | 30.6 |
| 2606 |  |  |  |  | 5 | 40 | 0.9 | 4.2 | 3.9 | 0.3 | 11.6 | 10.0 | 14.7 | 16.3 | 36.9 | 30.6 |
| 2608 |  |  |  |  | 8 | 30 | 0.9 | 4.2 | 3.9 | 0.3 | 11.6 | 10.0 | 14.7 | 16.3 | 36.9 | 30.6 |
| 2610 |  |  |  |  | 2 | 30 | 0.9 | 4.2 | 3.9 | 0.3 | 11.6 | 10.0 | 14.7 | 16.3 | 36.9 | 30.6 |
| 2612 |  |  |  |  |  |  | 0.9 | 4.2 | 3.9 | 0.3 | 11.6 | 10.0 | 14.7 | 16.3 | 36.9 | 30.6 |
| 2637 | SCL | 10YR5.6 | Yellowish brown | 20 | 2 | 7 |  | 3.6 | 3.3 | 0.3 | 12.0 | 11.0 | 9.9 | 33.6 | 16.3 | 21.4 |
| 2639 |  |  |  |  | 4 | 25 |  | 3.6 | 3.3 | 0.3 | 12.0 | 11.0 | 9.9 | 33.6 | 16.3 | 21.4 |
| 2641 |  |  |  |  | 2 | 20 |  | 3.6 | 3.3 | 0.3 | 12.0 | 11.0 | 9.9 | 33.6 | 16.3 | 21.4 |
| 2643 |  |  |  |  | 1 | 15 |  | 3.6 | 3.3 | 0.3 | 12.0 | 11.0 | 9.9 | 33.6 | 16.3 | 21.4 |
| 2645 |  |  |  |  | 2 | 5 |  | 3.6 | 3.3 | 0.3 | 12.0 | 11.0 | 9.9 | 33.6 | 16.3 | 21.4 |
| 2647 |  |  |  |  | 5 | 15 |  | 3.6 | 3.3 | 0.3 | 12.0 | 11.0 | 9.9 | 33.6 | 16.3 | 21.4 |
| 2689 |  |  |  |  | 3 | 15 |  | 3.6 | 3.3 | 0.3 | 12.0 | 11.0 | 9.9 | 33.6 | 16.3 | 21.4 |
| 2691 |  |  |  |  | 4 | 25 |  | 3.6 | 3.3 | 0.3 | 12.0 | 11.0 | 9.9 | 33.6 | 16.3 | 21.4 |
| 2692 |  |  |  |  | 2 | 5 | 1.3 | 3.7 | 1.1 | 0.1 | 7.8 | 3.7 | 6.0 | 13.4 | 19.2 | 44.0 |
| 2695 |  |  |  |  | 1 | 5 | 1.3 | 3.7 | 1.1 | 0.1 | 7.8 | 3.7 | 6.0 | 13.4 | 19.2 | 44.0 |
| 2696 |  |  |  |  | 2 | 15 | 1.3 | 3.7 | 1.1 | 0.1 | 7.8 | 3.7 | 6.0 | 13.4 | 19.2 | 44.0 |
| 2697 |  |  |  |  | 2 | 10 | 1.3 | 3.7 | 1.1 | 0.1 | 7.8 | 3.7 | 6.0 | 13.4 | 19.2 | 44.0 |
| 2698 |  |  |  |  | 1 | 5 | 1.3 | 3.7 | 1.1 | 0.1 | 7.8 | 3.7 | 6.0 | 13.4 | 19.2 | 44.0 |
| 2743 |  |  |  |  |  |  | 1.3 | 3.7 | 1.1 | 0.1 | 7.8 | 3.7 | 6.0 | 13.4 | 19.2 | 44.0 |
| 2775 |  |  |  |  | 7 | 40 | 1.3 | 3.7 | 1.1 | 0.1 | 7.8 | 3.7 | 6.0 | 13.4 | 19.2 | 44.0 |
| 2778 |  |  |  |  | 3 | 15 | 1.3 | 3.7 | 1.1 | 0.1 | 7.8 | 3.7 | 6.0 | 13.4 | 19.2 | 44.0 |
| 2780 |  |  |  |  | 3 | 5 | 1.3 | 3.7 | 1.1 | 0.1 | 7.8 | 3.7 | 6.0 | 13.4 | 19.2 | 44.0 |
| 2782 |  |  |  |  | 4 | 25 | 1.3 | 3.7 | 1.1 | 0.1 | 7.8 | 3.7 | 6.0 | 13.4 | 19.2 | 44.0 |
| 2784 |  |  |  |  | 2 | 7 | 1.3 | 3.7 | 1.1 | 0.1 | 7.8 | 3.7 | 6.0 | 13.4 | 19.2 | 44.0 |
| 2829 | MC | 10YR6.8 | Brownish yellow | 50 |  |  | 1.3 | 3.6 | 2.4 | 0.3 | 9.5 | 8.0 | 9.0 | 22.3 | 15.3 | 37.4 |
| 2831 |  |  |  |  |  |  | 1.3 | 3.6 | 2.4 | 0.3 | 9.5 | 8.0 | 9.0 | 22.3 | 15.3 | 37.4 |
| 2833 |  |  |  |  |  |  | 1.3 | 3.6 | 2.4 | 0.3 | 9.5 | 8.0 | 9.0 | 22.3 | 15.3 | 37.4 |
| 2835 |  |  |  |  |  |  | 1.3 | 3.6 | 2.4 | 0.3 | 9.5 | 8.0 | 9.0 | 22.3 | 15.3 | 37.4 |
| 2838 |  |  |  |  |  |  | 1.3 | 3.6 | 2.4 | 0.3 | 9.5 | 8.0 | 9.0 | 22.3 | 15.3 | 37.4 |
| 2875 | MC | 10YR6.6 | Brownish yellow |  | 30 |  | 1.1 | 3.5 | 3.7 | 0.3 | 12.5 | 10.0 | 12.3 | 14.1 | 20.3 | 34.2 |
| 2895 |  |  |  |  |  |  | 1.1 | 3.5 | 3.7 | 0.3 | 12.5 | 10.0 | 12.3 | 14.1 | 20.3 | 34.2 |
| 2897 |  |  |  |  |  |  | 1.1 | 3.5 | 3.7 | 0.3 | 12.5 | 10.0 | 12.3 | 14.1 | 20.3 | 34.2 |
| 2899 |  |  |  |  |  |  | 1.1 | 3.5 | 3.7 | 0.3 | 12.5 | 10.0 | 12.3 | 14.1 | 20.3 | 34.2 |
| 2970 | SCL | 10YR5.4 | Yellowish brown |  | 5 | 25 | 1.0 | 3.6 | 2.1 | 0.2 | 9.3 | 5.5 | 10.5 | 5.6 | 23.0 | 40.8 |
| 2972 |  |  |  |  | 3 | 35 | 1.1 | 3.6 | 1.7 | 0.2 | 8.9 | 5.3 | 9.6 | 11.1 | 27.8 | 33.9 |
| 2974 | LC | 10YR5.8 | Yellowish brown |  | 1 | 5 | 1.1 | 3.6 | 1.7 | 0.2 | 8.9 | 5.3 | 9.6 | 11.1 | 27.8 | 33.9 |
| 2976 |  |  |  |  | 1 | 1 | 1.1 | 3.6 | 1.7 | 0.2 | 8.9 | 5.3 | 9.6 | 11.1 | 27.8 | 33.9 |
| 2978 |  |  |  |  | 1 | 5 | 1.2 | 3.6 | 1.4 | 0.2 | 8.4 | 5.2 | 8.8 | 16.6 | 32.6 | 27.0 |
| 2996 |  |  |  |  | 1 | 5 | 1.1 | 3.6 | 1.7 | 0.2 | 8.9 | 5.3 | 9.6 | 11.1 | 27.8 | 33.9 |
| 2998 | LMC | 10YR7.8 | Yellow |  | 2 | 15 | 1.1 | 3.6 | 2.2 | 0.2 | 9.4 | 5.3 | 9.4 | 11.6 | 22.8 | 36.2 |
| 3010 |  | 10YR5.6 | Yellowish brown |  | 1 | 5 |  | 3.7 | 1.7 | 0.2 | 10.1 | 4.3 | 6.9 | 27.2 | 8.8 | 24.8 |
| 3012 |  |  |  |  | 2 | 60 |  | 3.7 | 1.7 | 0.2 | 10.1 | 4.3 | 6.9 | 27.2 | 8.8 | 24.8 |
| 3013 |  |  |  |  | 2 | 7 |  | 3.7 | 1.7 | 0.2 | 10.1 | 4.3 | 6.9 | 27.2 | 8.8 | 24.8 |
| 3014 |  |  |  |  | 1 | 5 |  | 3.7 | 1.7 | 0.2 | 10.1 | 4.3 | 6.9 | 27.2 | 8.8 | 24.8 |
| 3015 |  |  |  |  | 1 | 10 |  | 3.7 | 1.7 | 0.2 | 10.1 | 4.3 | 6.9 | 27.2 | 8.8 | 24.8 |
| 3060 | MC | 10YR5.6 | Yellowish brown |  | 2 | 5 |  | 3.8 | 2.0 | 0.2 | 9.5 | 5.2 | 7.9 | 20.0 | 10.0 | 21.6 |
| 3062 | LMC | 10YR7.6 | Yellow |  | 2 | 20 |  | 3.8 | 2.0 | 0.2 | 9.5 | 5.1 | 7.7 | 27.2 | 11.3 | 21.4 |
| 3063 | MC | 10YR6.8 | Brownish yellow |  | 2 | 15 |  | 3.8 | 2.0 | 0.2 | 9.5 | 5.1 | 7.7 | 27.2 | 11.3 | 21.4 |
| 3064 | LC | 10YR5.4 | Yellowish brown |  | 2 | 100 |  | 3.8 | 2.0 | 0.2 | 9.5 | 5.1 | 7.7 | 27.2 | 11.3 | 21.4 |
| 3066 | LMC | 10YR6.6 | Brownish yellow |  | 2 | 7 |  | 3.8 | 2.0 | 0.2 | 9.5 | 5.1 | 7.7 | 27.2 | 11.3 | 21.4 |
| 3067 | LMC | 10YR6.6 | Brownish yellow |  | 3 | 25 |  | 3.8 | 2.0 | 0.2 | 9.5 | 5.0 | 7.5 | 34.5 | 12.6 | 21.2 |
| 3068 | ZCL | 10YR5.4 | Yellowish brown |  | 2 | 15 |  | 3.8 | 2.0 | 0.2 | 9.5 | 5.1 | 7.7 | 27.2 | 11.3 | 21.4 |
| 3074 | LC | 10YR4.3 | Brown |  | 1 | 15 | 1.1 | 3.6 | 2.2 | 0.2 | 9.4 | 5.3 | 9.4 | 11.6 | 22.8 | 36.2 |
| 3076 | LC | 10YR5.8 | Yellowish brown |  | 1 | 10 | 1.1 | 3.6 | 2.2 | 0.2 | 9.4 | 5.3 | 9.4 | 11.6 | 22.8 | 36.2 |
| 3078 |  |  |  |  | 1 | 10 | 1.1 | 3.6 | 2.2 | 0.2 | 9.4 | 5.3 | 9.4 | 11.6 | 22.8 | 36.2 |
| 3079 | SC | 10YR6.8 | Brownish yellow |  | 1 | 5 | 1.1 | 3.6 | 2.2 | 0.2 | 9.4 | 5.3 | 9.4 | 11.6 | 22.8 | 36.2 |
| 3080 |  |  |  |  | 1 | 5 | 1.1 | 3.6 | 2.2 | 0.2 | 9.4 | 5.3 | 9.4 | 11.6 | 22.8 | 36.2 |
| 3081 | LC | 10YR5.8 | Yellowish brown |  | 1 | 5 | 1.1 | 3.6 | 2.2 | 0.2 | 9.4 | 5.3 | 9.4 | 11.6 | 22.8 | 36.2 |
| 3082 | LC | 10YR5.8 | Yellowish brown |  |  |  | 1.1 | 3.6 | 2.2 | 0.2 | 9.4 | 5.3 | 9.4 | 11.6 | 22.8 | 36.2 |
| 3083 | MC | 10YR6.8 | Brownish yellow |  | 1 | 7 |  | 3.8 | 1.9 | 0.2 | 9.6 | 4.1 | 6.7 | 44.2 | 13.0 | 18.8 |
| 3084 | MC | 10YR6.8 | Brownish yellow |  | 1 | 3 |  | 3.7 | 1.8 | 0.2 | 9.7 | 4.7 | 7.6 | 26.2 | 15.3 | 28.1 |
| 3085 | LC | 10YR5.6 | Yellowish brown |  |  |  |  | 3.7 | 1.7 | 0.2 | 9.8 | 5.3 | 8.5 | 8.2 | 17.6 | 37.4 |
| 3086 | LC | 10YR6.6 | Brownish yellow | 50 | 3 | 10 |  | 3.7 | 1.8 | 0.2 | 9.7 | 4.7 | 7.6 | 26.2 | 15.3 | 28.1 |
| 3087 | LMC | 10YR6.6 | Brownish yellow | 50 | 1 | 3 |  | 3.7 | 1.8 | 0.2 | 9.7 | 4.7 | 7.6 | 26.2 | 15.3 | 28.1 |
| 3088 | LC |  |  |  |  |  |  | 3.7 | 1.8 | 0.2 | 9.7 | 4.7 | 7.6 | 26.2 | 15.3 | 28.1 |
