# Supplementary material for: Site conditions for regeneration of climax species, the key for restoring moist deciduous tropical forest in Southern Vietnam
Source: PLoS One. 2020 May 29;15(5):e0233524. doi: 10.1371/journal.pone.0233524 (PMC7259571; doi:10.1371/journal.pone.0233524)
Supplement: S1 Table — (DOCX) [file pone.0233524.s003.docx]

**S1 Table. Characteristics of soil used in shade house experiment.**

| *Method* | *Nutrient* | *Units* | *Forest soil* | *Degraded soil* |
| --- | --- | --- | --- | --- |
| Bray1 | Phosphorus | mg/kg | 7.4 | 6.4 |
| Colwell |  |  | 24 | 24 |
| Bray2 |  |  | 8.1 | 7.4 |
| KCl | Nitrate Nitrogen | mg/kg | 3.9 | 4.3 |
|  | Ammonium Nitrogen |  | 30 | 24 |
|  | Sulfur |  | 20 | 60 |
| 1:5 Water | pH | units | 4.47 | 4.87 |
|  | Conductivity | dS/m | 0.070 | 0.044 |
| Calculation | Estimated Organic Matter | % OM | 3.1 | 1.5 |
| Ammonium Acetate + Calculations | Calcium | cmol^+^/Kg | 0.19 | 0.32 |
|  |  | kg/ha | 84 | 145 |
|  |  | mg/kg | 37 | 65 |
|  | Magnesium | cmol^+^/Kg | 0.26 | 0.13 |
|  |  | kg/ha | 70 | 36 |
|  |  | mg/kg | 31 | 16 |
|  | Potassium | cmol^+^/Kg | 0.22 | 0.15 |
|  |  | kg/ha | 191 | 134 |
|  |  | mg/kg | 85 | 60 |
|  | Sodium | cmol^+^/Kg | 0.02 | 0.03 |
|  |  | kg/ha | 11 | 17 |
|  |  | mg/kg | 5 | 7 |
| KCl | Aluminium | cmol^+^/Kg | 1.03 | 0.56 |
|  |  | kg/ha | 207 | 113 |
|  |  | mg/kg | 93 | 50 |
| Acidity Titration | Hydrogen | cmol^+^/Kg | 1.24 | 0.41 |
|  |  | kg/ha | 28 | 9 |
|  |  | mg/kg | 12 | 4 |
| Calculation | Effective Cation Exchange Capacity (ECEC) | cmol^+^/Kg | 2.95 | 1.61 |

**1**. All results presented as a 40°C oven dried weight. Soil sieved and lightly crushed to <2 mm

**2**. Methods from: Rayment, G. E., & Lyons, D. J. (2011). Soil Chemical Methods: Australasia. Australia: CSIRO Publishing.

**3**. Soluble Salts included in Exchangeable Cations - NO PRE-WASH

**4**. Total Acid Extractable Nutrients indicate a store of nutrient
