# Supplementary material for: Site conditions for regeneration of climax species, the key for restoring moist deciduous tropical forest in Southern Vietnam
Source: PLoS One. 2020 May 29;15(5):e0233524. doi: 10.1371/journal.pone.0233524 (PMC7259571; doi:10.1371/journal.pone.0233524)
Supplement: S2 Table — Values presented are means across each Bayesian model averaging model set. For each parameter, p ≠ 0 is the probability that the coefficient is not equal to zero. (DOCX) [file pone.0233524.s004.docx]

**S2 Table. Model-averaged coefficients for site and soil effect on *D. dyeri* seedling presence and abundance.** Values presented are means across each Bayesian model averaging model set. For each parameter, p ≠ 0 is the probability that the coefficient is not equal to zero.

|  | Parameters | p ≠ 0 | Coefficient | Standard deviation of coefficient |
| --- | --- | --- | --- | --- |
| Presence of seedlings | Intercept | 100 | 2.524 | 1.401 |
|  | BA | 39.4 | -0.01 | 0.016 |
|  | PD.A | 8.7 | 0.088 | 0.444 |
|  | pH | 6 | -0.008 | 0.258 |
|  | openness | 86.7 | -0.121 | 0.067 |
|  | Silt | 6.8 | -0.001 | 0.005 |
| Abundance of seedlings | Intercept | 100 | 1.795 | 2.554 |
|  | BA | 20.9 | 0.002 | 0.005 |
|  | PD.A | 15.8 | 0.122 | 0.359 |
|  | pH | 47 | -0.529 | 0.677 |
|  | openness | 10 | -0.002 | 0.01 |
|  | Silt | 23.4 | 0.003 | 0.007 |
| Presence of seedling on ME site | Intercept | 100 | 3.387 | 2.077 |
|  | BA | 97.5 | -0.074 | 0.029 |
|  | PD.A | 45.8 | 1.704 | 2.218 |
|  | pH | 7.3 | 0.068 | 0.398 |
|  | Silt | 4.3 | 0.0003 | 0.006 |
|  | openness | 23.2 | -0.028 | 0.063 |
|  | CEC | 72.7 | -0.419 | 0.356 |
|  | N | 31.9 | 4.608 | 17.861 |
|  | P_2_O_5_ | 55.8 | 0.307 | 0.334 |
| Abundance of seedling on ME site | Intercept | 100 | 5.702 | 2.435 |
|  | BA | 100 | 0.026 | 0.008 |
|  | PD.A | 7.4 | -0.018 | 0.176 |
|  | pH | 97.1 | -1.619 | 0.629 |
|  | Silt | 13.1 | 0.001 | 0.004 |
|  | openness | 9.4 | 0.002 | 0.011 |
|  | CEC | 8.2 | 0.001 | 0.03 |
|  | N | 11.8 | -0.328 | 1.37 |
|  | P_2_O_5_ | 12.7 | -0.006 | 0.022 |
| Presence of seedling on MB site | Intercept | 100 | -4.659 | 31.032 |
|  | pH | 17.7 | 1.289 | 6.250 |
|  | BA | 22.6 | -0.021 | 0.059 |
|  | TWI | 15 | 0.041 | 0.309 |
|  | openness | 15.6 | -0.007 | 0.041 |
|  | P2O5 | 15 | -0.179 | 3.132 |
|  | Silt | 17.6 | 0.019 | 0.095 |
