# Supplementary material for: Site conditions for regeneration of climax species, the key for restoring moist deciduous tropical forest in Southern Vietnam
Source: PLoS One. 2020 May 29;15(5):e0233524. doi: 10.1371/journal.pone.0233524 (PMC7259571; doi:10.1371/journal.pone.0233524)
Supplement: S1 Fig — Positive correlations are displayed in grey and negative correlations in black. The size of the circle are proportional of the correlation coefficients with the significant level 0.05. (DOCX) [file pone.0233524.s005.docx]

**S1 Fig. Correlation of soil and site properties at both sites (A), at ME only (B), at MB only (C), and at observation points where the seedling presented only (D).** Positive correlations are displayed in grey and negative correlations in black. The size of the circle are proportional of the correlation coefficients with the significant level 0.05.


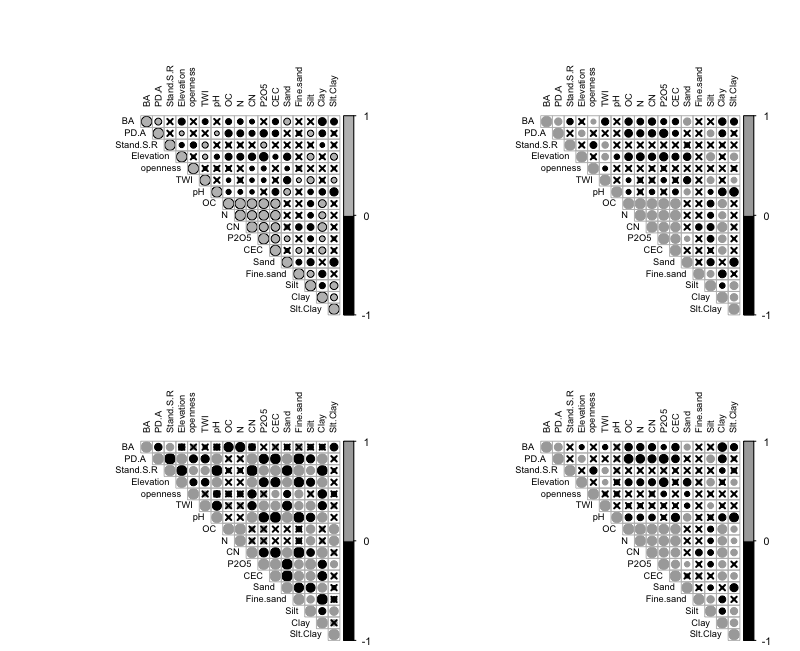


D

C

B

A
