# Supplementary material for: Site conditions for regeneration of climax species, the key for restoring moist deciduous tropical forest in Southern Vietnam
Source: PLoS One. 2020 May 29;15(5):e0233524. doi: 10.1371/journal.pone.0233524 (PMC7259571; doi:10.1371/journal.pone.0233524)
Supplement: S2 Fig — (DOCX) [file pone.0233524.s006.docx]

**S2 Fig 2. Fruit size (A), Branching of under-canopy seedling (B), Seedling died in shade**

(B)

**
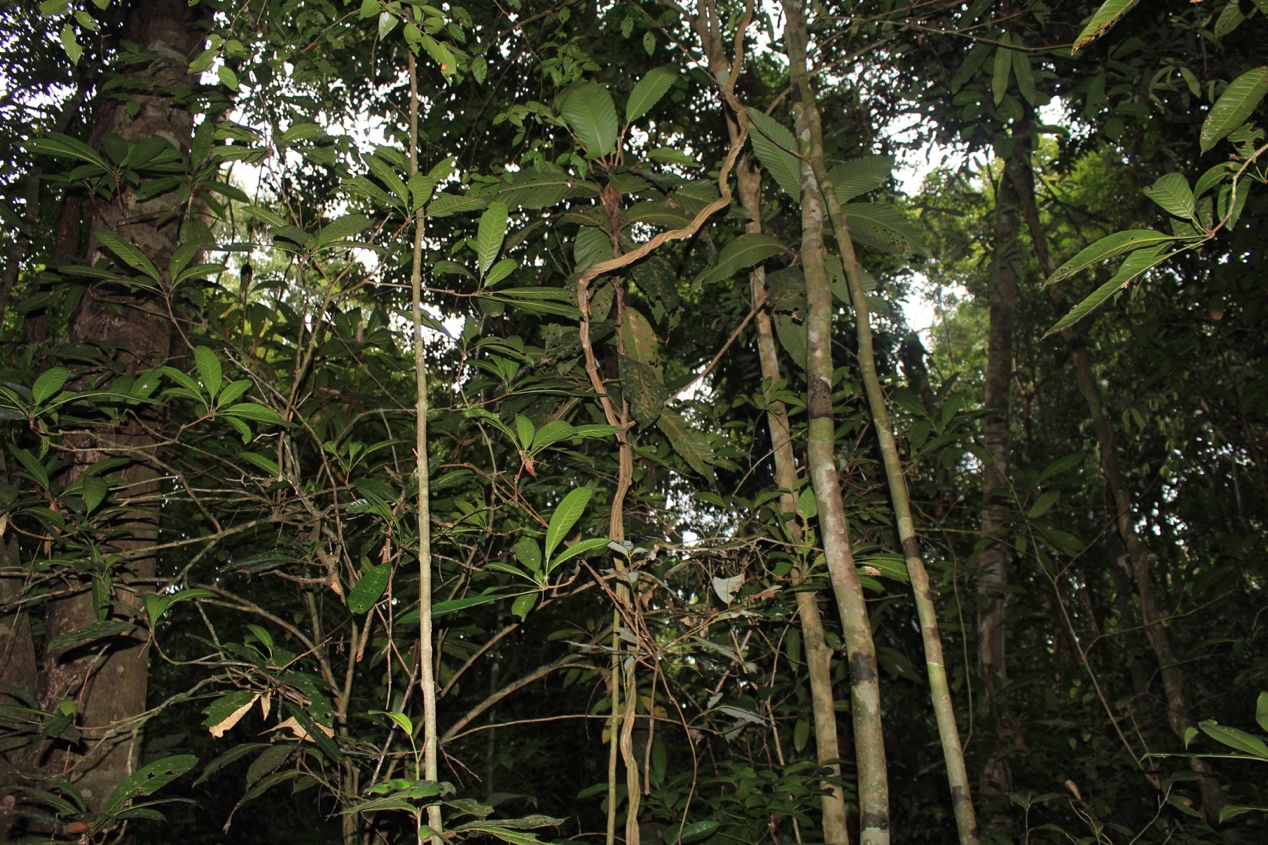

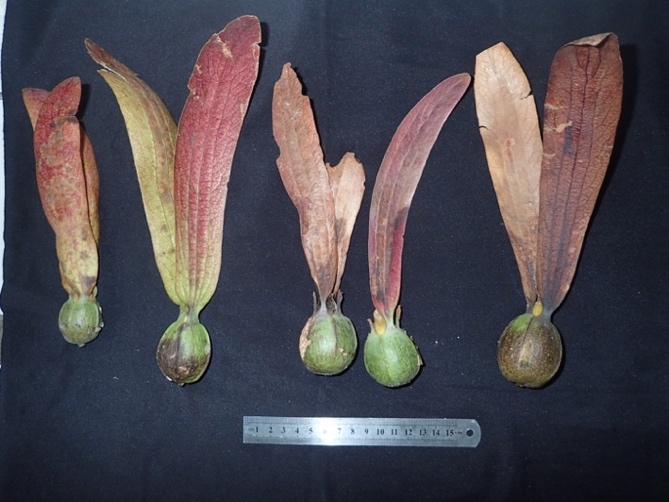
house and Fusarium samples (C)**


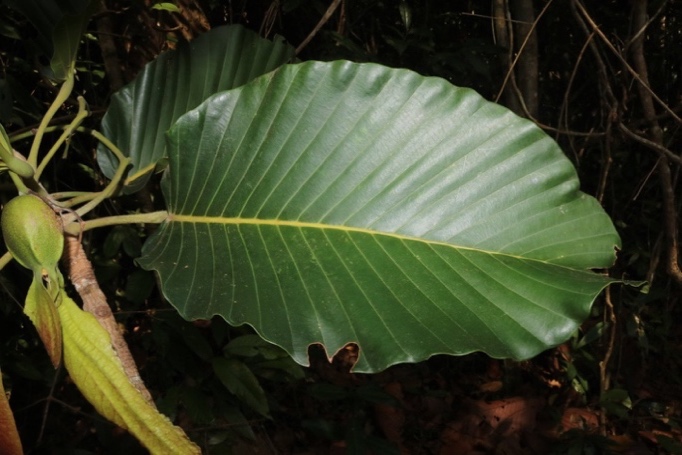


(B)

(A)


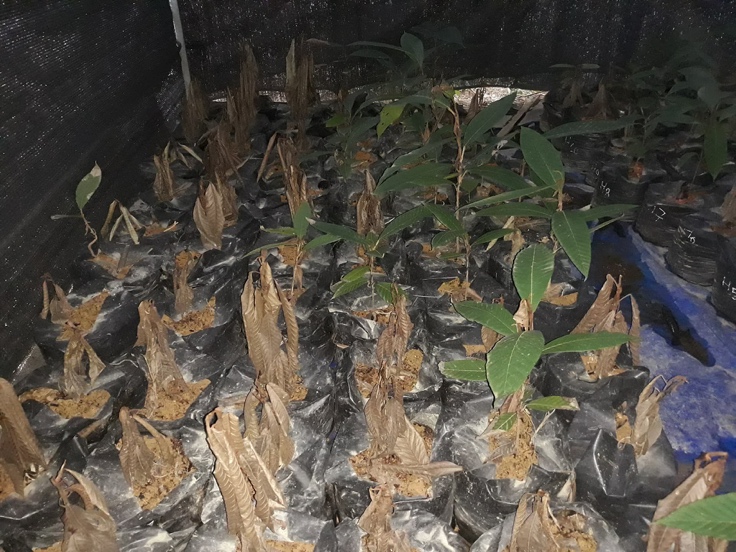

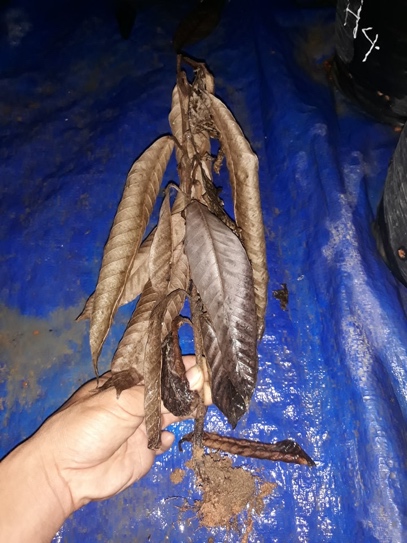


(C)

(E)


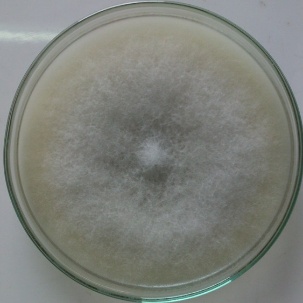

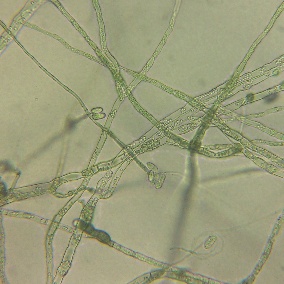

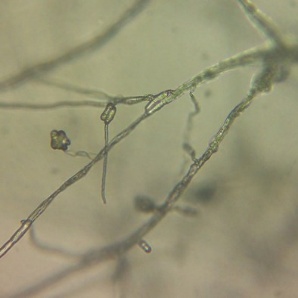


(D)
