# Supplementary material for: Site conditions for regeneration of climax species, the key for restoring moist deciduous tropical forest in Southern Vietnam
Source: PLoS One. 2020 May 29;15(5):e0233524. doi: 10.1371/journal.pone.0233524 (PMC7259571; doi:10.1371/journal.pone.0233524)
Supplement: Attachment — Submitted filename: Reviewers.docx [file pone.0233524.s007.docx]

**Manuscript Number:** PONE-D-19-35237
**Manuscript Title:** Site conditions for regeneration of climax species, the key for restoring moist deciduous tropical forest in Southern Vietnam

**Authors**: Ha Do1, John C. Grant1, Heidi C. Zimmer1, Ngoc-Bon Trinh, J. Doland Nichols

**Review comments:**

The authors of this paper have investigated the presence and abundance of *Dipterocarpus dyer*i seedlings across a range of environmental conditions, and performed an experiment in shade house to evaluate the effect of light levels on the mortality and health status of the seedlings of this species. The study is nevertheless interesting and is a necessary research work. The authors presented a reasonable methodology and results, and the paper in general is well written. However, there are a lot of typo errors and jumping off words, besides, some clarity on data collection and site information is missing. I suggest an acceptance after incorporation of these in the revised manuscript.

**Specific comments:**

L22. Abstract. Provide the level of significance

L25. Mention the % of mortality

L30-32. Sentences not clear. Briefly mention why recovery action is needed while *D. dyeri* is already a dominant species

L33. Provide keywords after the abstract.

L 85-86. Sentences not clear.

L99. Please give authority of the species (*Dipterocarpus dyeri*) used for the first time.

L106. Add a paragraph mentioning the treatment level of the species in South East Vietnam and causal factors associated to it

L110. Please provide the geo coordinates of the study sites

L117. 44 to 130 m asl???

L137. The authority of the species may be deleted

L146-154. What was the proximity between two sites (MB & ME sites)? How many 400m^2^ plots were established? What was the size of the other three plots (where no seedling of the target species, but the adult species found). Is it the same with as sampling clusters? Were there other plots too where no seedling got established? How such plots were compared? How many plots were considered for vegetation survey and for other site characteristics? Please mention.

L156. The methods of measurement of different variables that are included in the table-1 should be briefly included under the head ‘Data collection”.

L162-165. Mention how light levels were quantified. What was the experimental design and how many replicates were used for each treatment. How the seedlings were grown (in polypot???) and quantity soil added, and for how long the seedlings were allowed to grow in the shade house, need to be included.

L182. How canopy openness was measured while sampling was carried out in dry season where deciduous associates of D. dyeri shed their leaves?

L218 Expand the abbreviations used in Table 2 or please add the key as in table 1. The significant codes 0.05 ‘.’, 0.1 ‘ ‘ should be deleted as no such code is mentioned in the table.

L254 Provide seedling survival percent at medium and high light levels

L262 Table 3 Leaf damage may be put in parentheses after Health status for better clarity

L263. Expand all the abbreviations used as treatments in table 3.

L263. LSD value may be included against each column in Table 3.

L268. Influence to the seedlings be changed to influenced the seedlings

L269-270. Seedling grown in forested soil had slightly larger diameter? Compared to what?

Line 273. Based diameter should be replaced with base diameter

L276-279. The sentences may be rewritten for better clarity. Forest soil had greater leaves of leaf length??? Leaf wide may be replaced with leaf width.

L280-283. First time, results on leaf stomatal density and chlorophyll are mentioned. Please mention these in data collection under shade house component.

L298-299. May not necessarily be true always. Eg. Teak a large leaved species is a light demander.

L309. The results shows opposite trend in natural vs simulated (shade house) conditions; there may be possibilities that other factors (other than light) might have discernable influence on the presence or absence of seedling. The factors like litter thickness, soil CO_2_ concentration too would affect germination and process of seedling establishment. Please include these in the discussion.

L349. Typo errors. Please rectify the errors.

L363. Please specify if there was irrigation in the shade house as the level of irrigation would affect humidity, the result however would be different if there was controlled irrigation in the shade house

L381-388. This portion may be shifted to the conclusion part of the paper.

L390-394. These sentences may be shifted to the discussion part.
