# Supplementary material for: Site conditions for regeneration of climax species, the key for restoring moist deciduous tropical forest in Southern Vietnam
Source: PLoS One. 2020 May 29;15(5):e0233524. doi: 10.1371/journal.pone.0233524 (PMC7259571; doi:10.1371/journal.pone.0233524)
Supplement: Attachment — Submitted filename: Response to Reviewers.docx [file pone.0233524.s008.docx]

26th March 2020

Dear Editor:

RE: Revised manuscript PONE-D-19-35237

We are re-submitting the manuscript “*Site conditions for regeneration of climax species, the key for restoring moist deciduous tropical forest in Southern Vietnam*”” for consideration for publication as original article in the ***PLOS ONE*** **Biodiversity Conservation Collection***.* We have addressed the comments from the two reviewers and believe that the manuscript is much improved. These responses are detailed below. Reviewer comments are in Back_Arial and our responses are in Blue_Arial, the line numbers are in tracked changes (all Markup) version.

**We look forward to hearing from you in due course.**

**Yours truly,**

**Ha Do**

**On behalf of the authors.**

Review comments:

The authors of this paper have investigated the presence and abundance of *Dipterocarpus dyer*i seedlings across a range of environmental conditions, and performed an experiment in shade house to evaluate the effect of light levels on the mortality and health status of the seedlings of this species. The study is nevertheless interesting and is a necessary research work. The authors presented a reasonable methodology and results, and the paper in general is well written. However, there are a lot of typo errors and jumping off words, besides, some clarity on data collection and site information is missing. I suggest an acceptance after incorporation of these in the revised manuscript.

Specific comments:

Abstract. Provide the level of significance

L22. We added p values “Seedling presence (p = 0.065) and abundance varied significantly (p = 0.001) between the two sites,”

L25. Mention the % of mortality

L26-27. We add that “Seedling survival was significantly lower at the lowest light level (<10% full irradiance) at 13% for the forest soil and 25% for degraded soil. At higher irradiance the seedling survival rates were greater than 99%.”

L30-32. Sentences not clear. Briefly mention why recovery action is needed while *D. dyeri* is already a dominant species

L31-33. We have reworded and clarified the following sentences: “Historically, *Dipterocapus dyeri* was dominant in moist deciduous tropical forest across south-eastern Vietnam, but today it is rare. Active management of these recovering forests is essential in order to recover this high-value, climax forest species.

L33. Provide keywords after the abstract.

L35-36. We add “Keywords: canopy openness, *Dipterocarpus dyeri* Pierre, microsite, restoration, seasonally dry tropical forests, secondary forest”

L 85-86. Sentences not clear.

L95. We have reworded and clarified the following sentence: “Dipterocarpaceae was previously abundant throughout southeast Asia, however most remaining forests are heavily disturbed and are dominated by deciduous tree species and bamboo”

L99. Please give authority of the species (*Dipterocarpus dyeri*) used for the first time.

L115. We have added authority of the species: “*Dipterocarpus dyeri* Pierre”

L106. Add a paragraph mentioning the treatment level of the species in South East Vietnam and causal factors associated to it

L105-112. We add one paragraph: “Dipterocarpus dyeri is native to Myanmar, Thailand, Viet Nam, Cambodia and Peninsular Malaysia. The species has been globally assessed as Endangered in the IUCN Red list [53,69]. Most dipterocarp species, including Dipterocarpus dyeri Pierre, are high value timber species, and as such, have been selectively logged across South-east Asia [42]. Dipterocarpus dyeri has been targeted in particular because of its high density wood (~ 0.8 g/cm^3^) and also their resins (dammar) which can be extracted for caulking boats, varnish paint, and medicine [42,53–56]. *Dipterocarpus dyeri* today is one of the rarest species in the forest and there is limited information on species ecology regeneration and growing site conditions [53,57].”

L110. Please provide the geo coordinates of the study sites

L128-129. We have added coordinates of the Dong Nai Biosphere Reserve: “The Reserve extends between 11°20’50”N – 11°50’20”N and 107°09’05”E – 107°35’20”E”.

L117. 44 to 130 m asl???

L136. We have corrected this to: “130 m asl”.

L137. The authority of the species may be deleted

L156. We have deleted species authority.

What was the proximity between two sites (MB & ME sites)?

L167. We added “ approximately 20 km from ME site”

How many 400m^2^ plots were established?

L 165. We added: “from 18 sampling clusters (represented by plot data, Table 1, Fig 1, S1)”.

What was the size of the other three plots (where no seedling of the target species, but the adult species found). Is it the same with as sampling clusters?

L191. We added: “If none of seedlings was found, then a similar 400 m^2^ plot was established”.

Were there other plots too where no seedling got established?

L171-173. We added “If no seedlings were found, then a similar 400 m^2^ plot was established for surveying site and soil conditions, with a mother tree as the centre and five observation points (each of the four corners and at the centre of the plot)”.

How such plots were compared? How many plots were considered for vegetation survey and for other site characteristics? Please mention.

L164-165. “A total of 122 observation points (2 m x 2 m) were taken from 18 sampling clusters (represented by plot vegetation data, Table 1, Fig 1, S1).”

L156. The methods of measurement of different variables that are included in the table-1 should be briefly included under the head ‘Data collection”.

L174-197. We add method for data collection from forest of vegetation composition, light level and environment:

“**Vegetation composition.** In each plot, all woody species (excluding lianas) were recorded and divided into three classes based on the diameter at 1.3 m height from the ground (DBH): adults (DBH ≥ 10 cm), juveniles (5 cm ≤ DBH < 10 cm). Adults and juveniles were surveyed from 400 m^2^ plots while seedlings of all species were surveyed from the observation points.

Soil. At each observation point, a description of soil from 0-35 cm depth was made, including horizons, colour and texture. One topsoil (0-10 cm depth) sample which was a mixture of soil from 3-5 observation points was taken for soil chemical characterisation of each sampling cluster. At each sampling cluster one core sample (5 cm diameter x 5 cm length) was taken for topsoil bulk density calculation.

“Light. A hemispherical canopy photograph was taken at each observation point at 170 cm above the ground. We chose this height to avoid the shrub and herb layers. These photos were used to determine canopy openness (%) using Gap Light Analyzer (GLA) software [1], after processing the image through SideLook [2] using automatic thresholding. The procedure of using automatic threshold to adjudge colour canopy photographs is recommended for assessing canopy openness [3].”

Environment. Basic site conditions were described at each observation point, including slope (degrees), aspect (degrees), outcrop rock (% cover), forest floor vegetation cover (%), litter cover (%) and litter thickness (cm). Topographic wetness index (TWI) [4], was calculated as a soil moisture indicator [5,6] for each observation point was derived by Arctoolbox from a digital elevation model (DEM) through SAGAGIS, AcrGIS v 10.1. A DEM of 30 x 30 m grid spacing was downloaded from <https://earthexplorer.usgs.gov/>.”

L162-165. Mention how light levels were quantified. What was the experimental design and how many replicates were used for each treatment. How the seedlings were grown (in polypot???) and quantity soil added, and for how long the seedlings were allowed to grow in the shade house, need to be included.

L198-212. We have added detail on how the shade house experiment was established, including measurement of light levels:

“Shade house experiment establishment

Seeds were collected from one mother tree from Cay Gui Forest Ranger Station (approximately 500 m from plot CG1). The seedlings were germinated using moist sand for 20-30 days until developing to seedlings with two true leaves (Figure A.2 B) which were then transplanted in plastic pots (8 cm diameter x 40 cm height). There were six treatments of three light levels (high, medium and low) and two sources of soil (old growth forest and heavily logged and degraded forest) with a total of 432 seedlings (72 seedlings per treatment). Each light treatment was set up in a shelter with light levels controlled by shade cloth, one layer for the high level, doubling for the medium level and tripling for the low level. The light levels were confirmed by the measurement of leaf area index (LAI) by using Li-2200. The LAI at the three shelters were 2.9, 5.4 and 7.1 giving estimations of canopy openness for each light levels were proximately 45%, 20% and 5% of full sun, respectively. Soil used for the trial was the topsoil of a Chromic Acrisol (Fp) and it was collected from two sites. Both soils were light clays in texture and the old growth forest soil (F) was higher in organic matter, total nitrogen and CEC than the degraded site soil (D) (Appendix 2).”

L182. How canopy openness was measured while sampling was carried out in dry season where deciduous associates of D. dyeri shed their leaves?

L186-191. We added light level measurement and analysis canopy openness:

“Light. A hemispherical canopy photograph was taken at each observation point at 170 cm above the ground. We chose this height to avoid the shrub and herb layers. These photos were used to determine canopy openness (%) using Gap Light Analyzer (GLA) software [1], after processing the image through SideLook [2] using automatic thresholding. The procedure of using automatic threshold to adjudge colour canopy photographs is recommended for assessing canopy openness [3].”

Expand the abbreviations used in Table 2 or please add the key as in table 1. The significant codes 0.05 ‘.’, 0.1 ‘ ‘ should be deleted as no such code is mentioned in the table.

L270-271. We have added the abbreviations: “ME: Mixed evergreen, MB: Mixed bamboo, BA: Stand basal area, PD.A: Dominance of adult *D. dyeri”*

L272. We have deleted the significance codes.

Provide seedling survival percent at medium and high light levels

L308. We added seedling survival percent ”(99% survival, only one seedling died in F.M treatment)”

Table 3 Leaf damage may be put in parentheses after Health status for better clarity

L319 – Table 3. We have corrected this to read: “ Health status (Leaf damage)”

L263. Expand all the abbreviations used as treatments in table 3.

L317. We have added abbreviations: “F: Forested soil, D: degraded soil, H: high light level, M: medium light level, L: Low light level”

L263. LSD value may be included against each column in Table 3.

As table 3 reports the results of a Chi squared test it is not appropriate to include least significant values (these are commonly reported alongside ANOVA tables); however we have added the LSD values associated with the results of the ANOVA, reported in Figure 3 (L322-332): “ANOVA results showed that light level, soil and light: soil interaction significantly influenced seedling height (p = 0.03; Fig 3), however, only light level resulted in a significant difference in seedling diameter (p =0.000; Fig 3). The seedlings grown at a high light level grew higher (Low – High =-37.1, p = 0.000; Medium - High = -26.35, p = 0.000, Medium – Low - 10.75, p =0.001) and larger (Low – High =-1.88, p = 0.000; Medium - High = -2.06, p = 0.000, Medium – Low = -0.19, p =0.73) than those in low light levels (Fig 3). Soils significantly influenced the seedling height at the high light level treatment (p=0.000; Fig 3A). The seedlings grown on forest soil had significantly higher stem height (Forest-Degraded = 6.39, p =0.000; Fig 3A) and slightly larger diameters than those grown in degraded soil (Forest-Degraded =0.15, p=0.29; Fig 3B) and. The seedlings grown at low and medium light levels were very similar in growth with no difference between the two soils.

We have also improved Fig 3 by presenting the results from ANOVA test and LSD tests.

L268. Influence to the seedlings be changed to influenced the seedlings

L328. We have changed this phrase to read: “Soils significantly influenced the seedling height”

L269-270. Seedling grown in forested soil had slightly larger diameter? Compared to what?

L330. We have added: “than those grown in degraded soil”

Line 273. Based diameter should be replaced with base diameter

L334. We have changed to: “Fig 3. Seedlings growth in base diameter (A), and height (B).”

L276-279. The sentences may be rewritten for better clarity. Forest soil had greater leaves of leaf length??? Leaf wide may be replaced with leaf width.

L338-339. We have improved the sentence :“Seedlings in higher light and on forest soil had greater leaves of leaf length (p < 0.001) and leaf width (p <0.001) (Fig 4 A, B) but thinner leaf thickness (Fig 4C)”

L280-283. First time, results on leaf stomatal density and chlorophyll are mentioned. Please mention these in data collection under shade house component.

L336-337. We added in the beginning of the paragraph: “The data on seedling leaf characteristics after 10 months was collected from shade house component of three light levels and two soil type”

L298-299. May not necessarily be true always. Eg. Teak a large leaved species is a light demander.

L363. We changed to “Seedlings which have high leaf area, such as *D. dyeri*, are more likely disadvantaged in large gaps”.

L309. The results shows opposite trend in natural vs simulated (shade house) conditions; there may be possibilities that other factors (other than light) might have discernable influence on the presence or absence of seedling. The factors like litter thickness, soil CO_2_ concentration too would affect germination and process of seedling establishment. Please include these in the discussion

L430-437. We added this point to our discussion “ Seedling mortality was greatest in the lowest light level in the shade house trial (Table 3). The results show opposite trends in natural vs simulated (shade house) conditions; it is possible that factors other than light might have discernible influence on the presence or absence of seedlings. Local site temporal factors such as litter thickness, soil CO_2_ concentration, presence of herbivores and pathogens can affect germination and process of seedling establishment. In our case, there was the evidence of *Fusarium* wilt disease (S2 Fig 2C), which infected the plants in the lower light and higher humidity conditions. In addition, while all seedlings were given the same amount of irrigation the soils in heavy shade appeared to stay wetter.”

L349. Typo errors. Please rectify the errors.

L417. We have corrected this: “, the higher fertility soils can be found on sloping sites“

L363. Please specify if there was irrigation in the shade house as the level of irrigation would affect humidity, the result however would be different if there was controlled irrigation in the shade house

L436-437. We added “while all seedlings were given the same amount of irrigation but soils in heavy shade appeared to stay wetter”.

L381-388. This portion may be shifted to the conclusion part of the paper.

L468-500. We have moved this section to the conclusion, as suggested: “With regard to restoration techniques, our findings suggest that (a) nurse crops are not necessary for *D. dyeri* establishment, if the site is moist enough, but (b) nurse crops should be used at drought-prone sites; (c) under the most common tree cover conditions, including intermediate recovered forest or dense plantation, light liberation will be necessary for native forest restoration; (d) restoration should not be attempted on waterlogged sites and (e) using phosphorus or lime carefully to increase soil phosphorus (but maintaining pH < 5.5) should be considered.”

L390-394. These sentences may be shifted to the discussion part.

L379-382. We moved to discussion “This is consistent with the findings of Denslow [11] and Paoli et al. [92] who found that in rainforest, most canopy species are neither open environment pioneer species nor shade-preferring “climax” forest species but generalists that can tolerate low light but require more open condition to grow into canopy trees [93–95].”
